# The predictability of fluctuating environments shapes the thermal tolerance of marine ectotherms and compensates narrow safety margins

**DOI:** 10.1038/s41598-024-77621-1

**Published:** 2024-10-30

**Authors:** Marco Fusi, Alberto Barausse, Jenny Marie Booth, Erica Chapman, Daniele Daffonchio, William Sanderson, Karen Diele, Folco Giomi

**Affiliations:** 1 Dove Marine Laboratory, School of Natural and Environmental Sciences, Newcastle University, Newcastle, NE1 7RU UK; 2 King Abdullah University of Science and Technology (KAUST), Red Sea Research Center (RSRC), 23955-6900 Thuwal, Saudi Arabia; 3 Centre for Conservation and Restoration Science, Edinburgh Napier University, Sighthill Campus, Edinburgh, UK; 4 Department of Biology, University of Padua, Via U. Bassi 58/B, 35131 Padua, Italy; 5 Department of Industrial Engineering, University of Padova, Via Gradenigo 6/a, 35131 Padua, Italy; 6 National Biodiversity Future Center (NBFC), Piazza Marina 61, 90133 Palermo, Italy; 7 Joint Nature Conservation Committee, Quay House, 2 East Station Road, Fletton Quays, Peterborough, PE2 8YY UK; 8 Coastal Research Group, Department of Zoology and Entomology, Rhodes University, PO Box 94, Grahamstown, 6140 South Africa; 9 St Abbs Marine Station, St Abbs, UK; 10 Centre for Marine Biodiversity and Biotechnology, ILES, EGIS, Heriot-Watt University, Edinburgh, UK; 11 School of Applied Science, Edinburgh Napier University, Sighthill Campus, Edinburgh, UK; 12 Department of Biology, University of Rome Tor Vergata, Rome, Italy

**Keywords:** Biogeography, Circadian cycle, Climate sensitivity, Macro-physiology, Phenotypic plasticity, Ecology, Physiology, Ecology

## Abstract

Aquatic species living in productive coastal habitats with abundant primary producers have evolved in highly dynamic diel and seasonally fluctuating environments in terms of, for example, water temperature and dissolved oxygen. However, how environmental fluctuations shape the thermal tolerance of marine species is still poorly understood. Here we hypothesize that the degree of predictability of the diel environmental fluctuations in the coastal area can explain the thermal response of marine species. To test this hypothesis, we measured the thermal tolerance of 17 species of marine ectotherm from tropical, warm temperate and cold temperate latitudes under two levels of oxygen (around saturation and at supersaturation), and relate the results to their site-specific temperature and oxygen fluctuation and their environmental predictability. We demonstrate that oxygen and temperature fluctuations at tropical latitudes have a higher predictability than those at warm and cold temperate latitudes. Further, we show that marine species that are adapted to high predictability have the potential to tune their thermal performance when exposed to oxygen supersaturation, despite being constrained within a narrow safety margin. We advocate that the predictability of the environmental fluctuation needs to be considered when measuring and forecasting the response of marine animals to global warming.

## Introduction

Productive aquatic environments with abundant primary producers are characterized by diel fluctuations of oxygen, determined by the cyclical succession of photosynthesis and community respiration, driven by the periodicity of light availability and temperature variations^1^. The partial co-dependency of the diel variation of dissolved oxygen on water temperature determines cyclical fluctuations which are approximately in phase^2^. Increasing the spatial and temporal resolution of observations reveals a complex pattern of variability in magnitude and frequency of the oscillations of these environmental drivers, including the occurrence of cyclical and stochastic fluctuations^3–5^. For example, there is a large daily variability in dissolved oxygen across marine environments, including a pervasive, but generally neglected, occurrence of oxygen supersaturation and its synchrony with changes in water temperature^6–8^.

Aquatic species that have evolved in dynamic, fluctuating environments and, especially in coastal zones or in other productive marine habitats, are adapted to endure large daily variations in oxygen and temperature^9^. However, the effect of these fluctuations on animal physiology remains poorly understood, because only a few studies have been designed with an ecologically relevant experimental design^9–11^. Nevertheless, it has been demonstrated that the large diel fluctuations of dissolved oxygen in tropical coastal ecosystems shape the resistance and resilience of associated fauna to warming. Specifically, oxygen supersaturation conditions during daylight increase the thermal tolerance and improve the physiological response of species living in shallow water ecosystems during nocturnal hypoxia^6^. Increased thermal tolerance in aquatic ectotherms exposed to large fluctuations of dissolved oxygen is not limited to tropical environments, but also occurs in productive aquatic habitats across other latitudes^7,12^. Similarly, daily and seasonal temperature fluctuations promote physiological and behavioural plasticity in wild fish, increasing their thermal response and tolerance^13^.

Organisms respond to environmental fluctuation through two ways, so-called feedback and feedforward phenotypic plasticity^1^. The former is characteristic of organisms that perceive changes in the environment and respond with physiological and behavioural adjustments that tend to minimize severe negative consequences. For example, marine ectotherms exposed to warming produce heat shock proteins to reduce cellular damage and physiological disruption^13,14^. On the other hand, feedforward phenotypic plasticity is exhibited by organisms exposed to cyclical environmental variations (i.e., regular periodic fluctuations), enabling them to anticipate environmental changes by tuning physiological and behavioural traits. For example, *Cerithidea decollata*, an intertidal mangrove climbing snail, can predict the onset of high tide, climbing up the mangrove trunk to avoid submersion and predation^15^. Numerous studies have addressed feedback and feedforward phenotypic plasticity of marine ectotherms to environmental stresses (e.g.,^16,17^), but new studies shed light on novel organismal response across latitudes, especially when considering relevant ecological conditions^9^.

Here we investigate the influence of environmental fluctuation on the thermal tolerance of several marine coastal species and we tentatively interpret the results in the light of environmental predictability (i.e., the regularity of these environmental oscillations). Specifically, we measured and compared the effect of temperature and oxygen fluctuations on the thermal response of coastal ectotherms across latitudes, hypothesizing that the magnitude of environmental predictability influences their heat tolerance and thermal safety margins (i.e., the tolerable excess of heat with respect to the typical local environmental conditions) and consequently tunes their plastic response. To test this hypothesis, we collected timeseries of oxygen and temperature from productive coastal aquatic habitats from three climatic regions (i.e., tropical, warm temperate, and cold temperate) and analysed the thermal response of associated fauna at different levels of oxygen saturation.

## Results

**Fig. 1:**
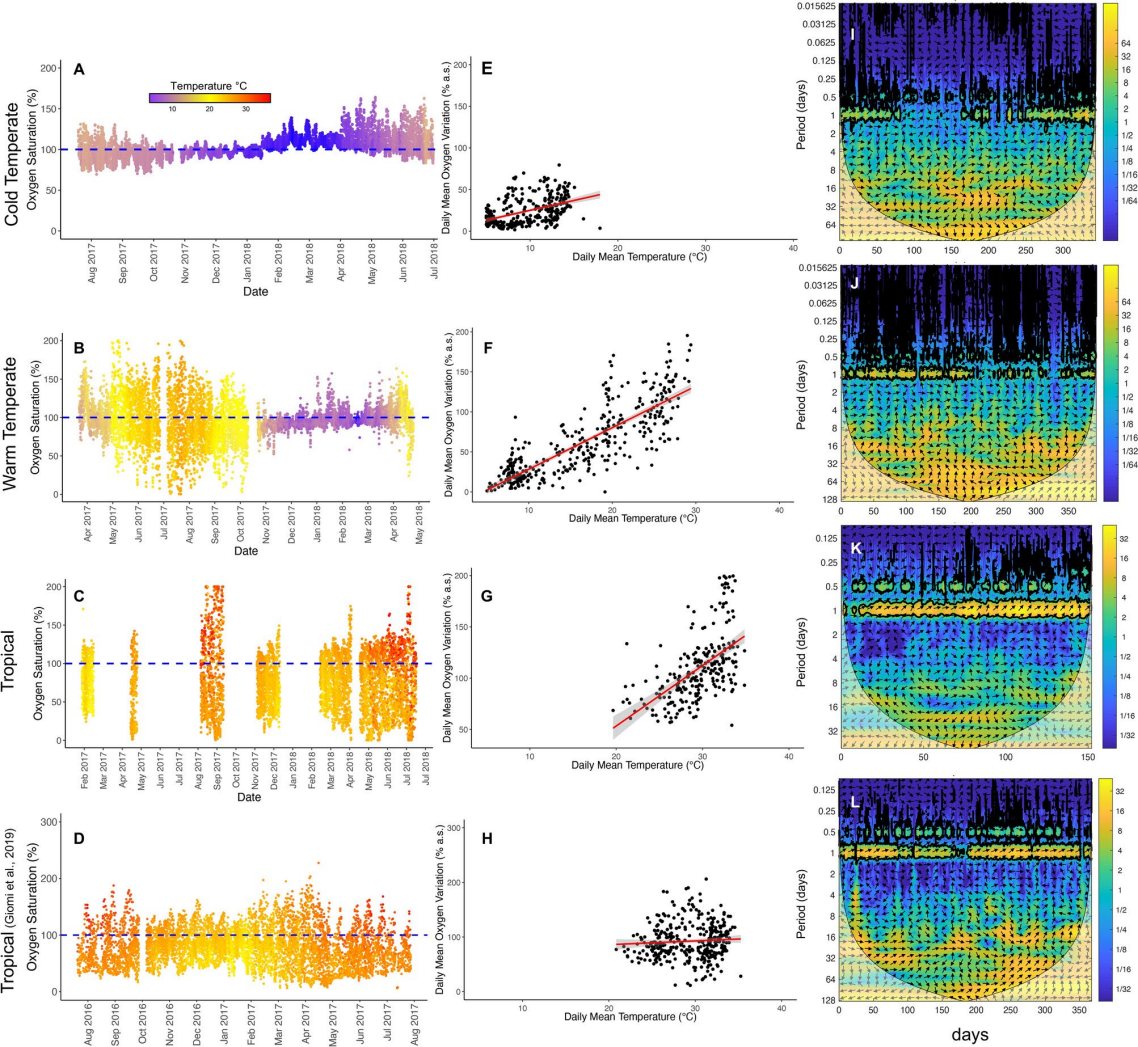
(**A**–**D**) Seawater oxygen saturation and temperature in the Cold Temperate, Warm Temperate and Tropical coastal sites (see Supplementary figure S4 for details on temperature variations). (**E**–**H**) Increase of the daily mean oxygen variation in function of the daily mean temperature calculated on the detrended time series; the red line indicates the trend of such correlations where the shaded area represents the 95% interval of confidence; %a.s. = air saturation. (**I**–**L**) Cross-wavelet analysis showing the relationship between oxygen and temperature in the three study sites. The horizontal axis represents time, while the vertical axis represents the periodicity of the corresponding oscillation. The paler area is the cone of influence, where results should not be interpreted due to biases connected to edge effects, and the thick black contour is the 5% significance level. The colour scale indicates the power of the cross-wavelet. Arrows represent the relative phase of the time series (pointing left when the two time series are in antiphase, right when in-phase). For the tropical site, the data set from Giomi et al., 2019^6^ is also included (**D**,**H**,**L**).

The three sites have three radically different annual temperature regimes, reflecting the climate zone in which they are located (Supplementary table S1A–D; Fig. S1; Fig. S4). The Cold Temperature site has a thermal regime that ranges from 4 to 15 °C with peaks up to 20 °C (Fig. 1A; Fig. S1), the Warm Temperate site has a wider range from close to 2 °C to 35 °C (Fig. 1B; Fig. S1), whereas the Tropical site has consistently higher temperatures around 30 °C with peaks of 41 °C in the hot period and lower than 20 °C in the colder period (Fig. 1C; Fig. S1). This is paralleled by the oxygen saturation regimes. All three sites showed fluctuations of oxygen from low to high levels of saturation but with a significant change in their typical value as well as temporal occurrence throughout the year (Fig. S3). In the Cold Temperate site there is a high frequency of oxygen saturation measurements around 100%, whereas in the Warm Temperate site the oxygen saturation frequency peaks around 80%. The Tropical site shows a more even frequency distribution of oxygen saturation, reflecting stronger daily fluctuations regularly occurring throughout the year (Fig. 1C,D, Figs. S1, S3). This is explained by a highly significant correlation between the detrended time series of the daily oxygen saturation variation and daily mean temperature. The relationship was highest with a 1 h time lag in the Cold Temperate and Tropical sites (Pearson r = 0.15 and 0.16, respectively; P < 0.001) and at a 0 h lag for the Warm Temperate site (r = 0.24, P < 0.001). This indicates that there is a strong diel synchronicity between temperature and oxygen over a large latitudinal range.

Analysis of temperature as an explanatory variable to explore the fluctuation of oxygen (Fig. S1) shows that the increase in temperature is paralleled by an increase in the oxygen range above and below 100% saturation, with consistent supersaturation associated to the Tropical site at higher temperatures, whereas in the Cold Temperate site water can be supersaturated at intermediate temperatures but not at higher temperatures (Fig. 1E–H, Fig. S1).

### Cross-wavelet analysis and environmental predictability

We computed the continuous cross-wavelet analysis to identify the dominant periodicities of the covariance of temperature and oxygen time series and to determine whether these were regular, periodic oscillations or isolated or intermittent bursts. All the study sites were characterized by regular daily oscillations in water temperature and dissolved oxygen (Fig. S3), consistently covering more than about half of the investigated period (Table 1). These fluctuations were particularly regular and predictable in the Tropical site, where they were present approximately 90% of the time. The cross-wavelet analysis clearly showed that the daily oscillations of dissolved oxygen and water temperature co-varied approximately in phase over time, and an additional weaker co-variance emerged at a semi-diurnal time scale presumably linked to tides (Fig. 1I–L).

**Table 1:** Predictability of the temperature (T) or oxygen (DO) fluctuations characterized by a periodicity of 24 h in the three sites studied. For the Tropical site we also used the data set from Giomi et al., 2019^6^ marked with the asterisks.

Signal and site	Days with a statistically significant 24 h periodicity/total monitored days not covered by the cone of influence	Predictability of daily fluctuations (% of time outside of the cone of influence with a statistically significant 24 h periodicity)
T—cold temperate	168/341	49%
DO—cold temperate	242/341	71%
T—warm temperate	193/389	50%
DO—warm temperate	240/389	62%
T—tropical	133/150–339/364*	89–93%*
DO—tropical	136/150–332/364*	91–91%*

### Animal survival

**Fig. 2:**
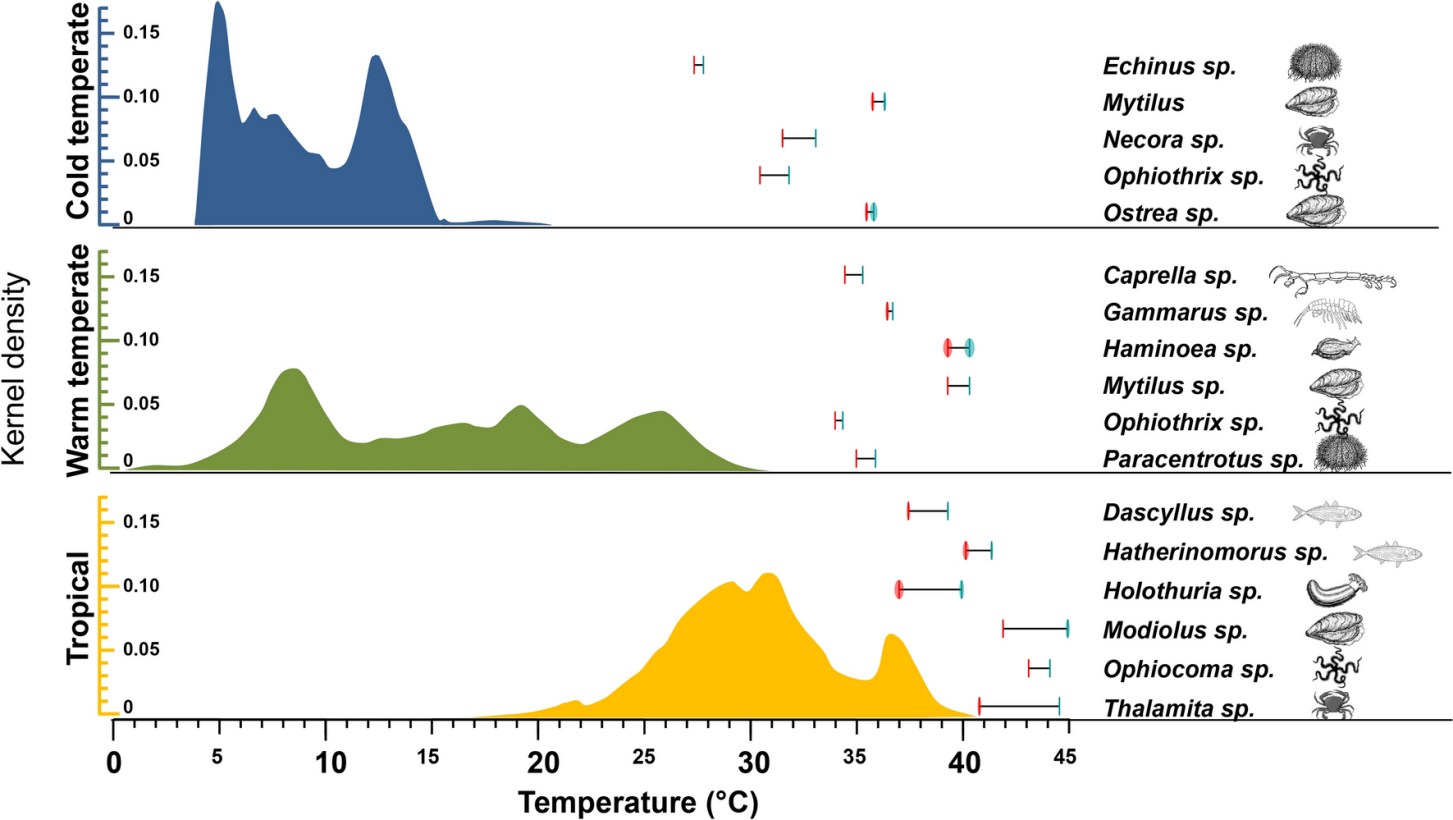
Temperature regimes and species-specific thermal tolerance of marine organisms at three different latitudes. On the left, frequency distributions of seawater temperature in the Cold Temperate, Warm Temperature and Tropical coastal sites from February 2017 to August 2018 are shown. On the right, thermal tolerance of 17 marine species (6 phyla) measured as lethal temperatures (LT50) of individuals exposed at oxygen full saturation (red symbols) and supersaturation (cyan symbols) are shown. The size of the symbols represents the standard error of the average (n = 6 to 44), while the length of the black bar connecting the symbols indicates the net gain in tolerance between the two oxygen levels. All the animal exposed to oxygen supersaturation extended their thermal tolerance in all three locations, but to a larger extent at the tropical than at the extratropical latitudes.

All the marine ectotherms tested by warming whilst exposed to experimentally induced oxygen supersaturation (140 ± 3% of oxygen saturation) had significantly enhanced lethal heat thresholds, extending their survival under high temperatures (Fig. 2 and Fig. S2; Supplementary tables S2, S3, S4, S6). However, the animals responded differently according to the species and the location tested^6^. The LT50 of the species exposed to oxygen supersaturation tested in the Tropical site increased by 1° to 4 °C, compared to full saturation (97 ± 2% of oxygen saturation). Warm Temperate specimens showed a narrow range of increased heat tolerance with an increase of 0.5° up to 2 °C, whereas heat tolerance of Cold Temperate species increased by 1° up to approximately 2.5 °C.

### Thermal safety margins

**Fig. 3:**
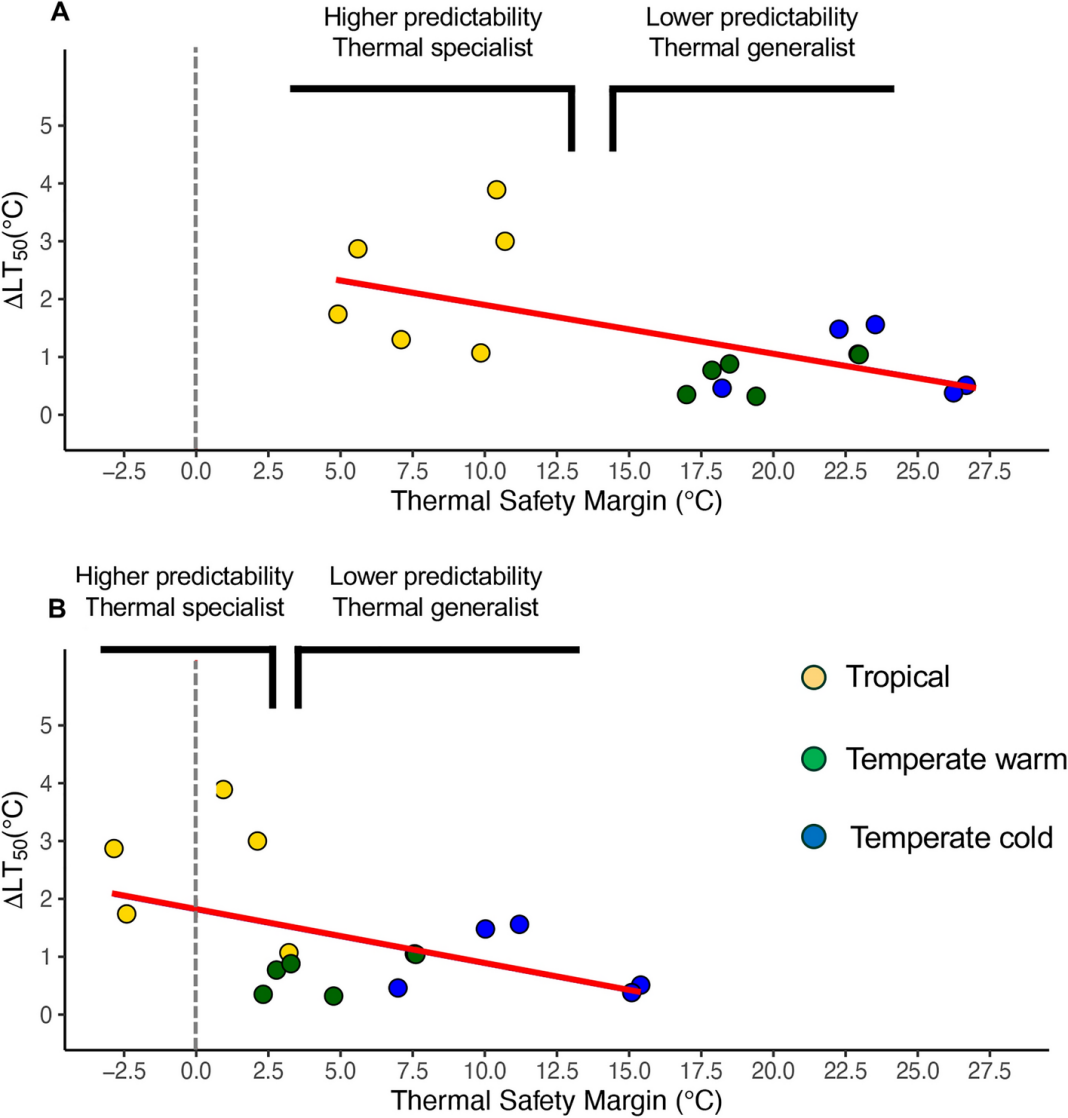
Relationship between ΔLT50 (LT50 at supersaturation – LT50 at full saturation) and Thermal Safety Margins. Difference between the heat tolerance of animals exposed to oxygen supersaturation and full saturation as function of the thermal safety margins, estimated as the difference between the LT50 under supersaturation (**A**) and full saturation of oxygen (**B**), and the daily average of maximum environmental temperatures measured in the three sites studied. Note that the thermal safety margin calculated with LT50 at full saturation are not strictly ecologically relevant since the highest temperatures occur only in conditions of supersaturation; this relationship is shown only for comparison. Red line represents the regression line from the linear model used to test this relationship and the grey dashed line represents the 0 Thermal safety margin. The gain of heat tolerance due to oxygen supersaturation is higher when the thermal safety margin is lower. Furthermore, the species that have the higher gain are living in highly predictable environments compared to those with lower gain living in less predictable environments. Red dashed line indicates when the thermal safety margin is 0. The black segments on the top identify the points with higher or lower predictability (see Table 1).

We found a significant negative relationship (*y* = 2.68–0.087*x*; F_1,15_ = 12.23; Adjusted R^2^: 0.41 p < 0.001) between the thermal safety margins of each species (calculated considering the average maximum temperature at each location) and their respective ΔLT50 (calculated as the difference between the lethal temperature at oxygen supersaturation and full saturation). Higher ΔLT50 was significantly correlated with lower thermal safety margins, the lowest having been identified in our study at the Tropical site; vice versa, the lower ΔLT50 were associated with the species living at higher latitude exposed to lower temperature, with higher thermal safety margins^18^ (Fig. 3A). Likewise, the same significant negative relationship was confirmed when the thermal safety margin was calculated between the highest temperature and the LT50 under 100% oxygen saturation at each location (*y* = 1.82–0.093*x*; F_1,15_ = 4.75; Adjusted R^2^: 0.19; p < 0.05); although for tropical species we observed the occurrence of negative safety margins if oxygen supersaturation was not taken into account when calculating the LT50 (Fig. 3B). Indeed, for tropical species we observed that the maximum temperatures consistently corresponded to high oxygen levels in productive aquatic environments.

**Fig. 4:**
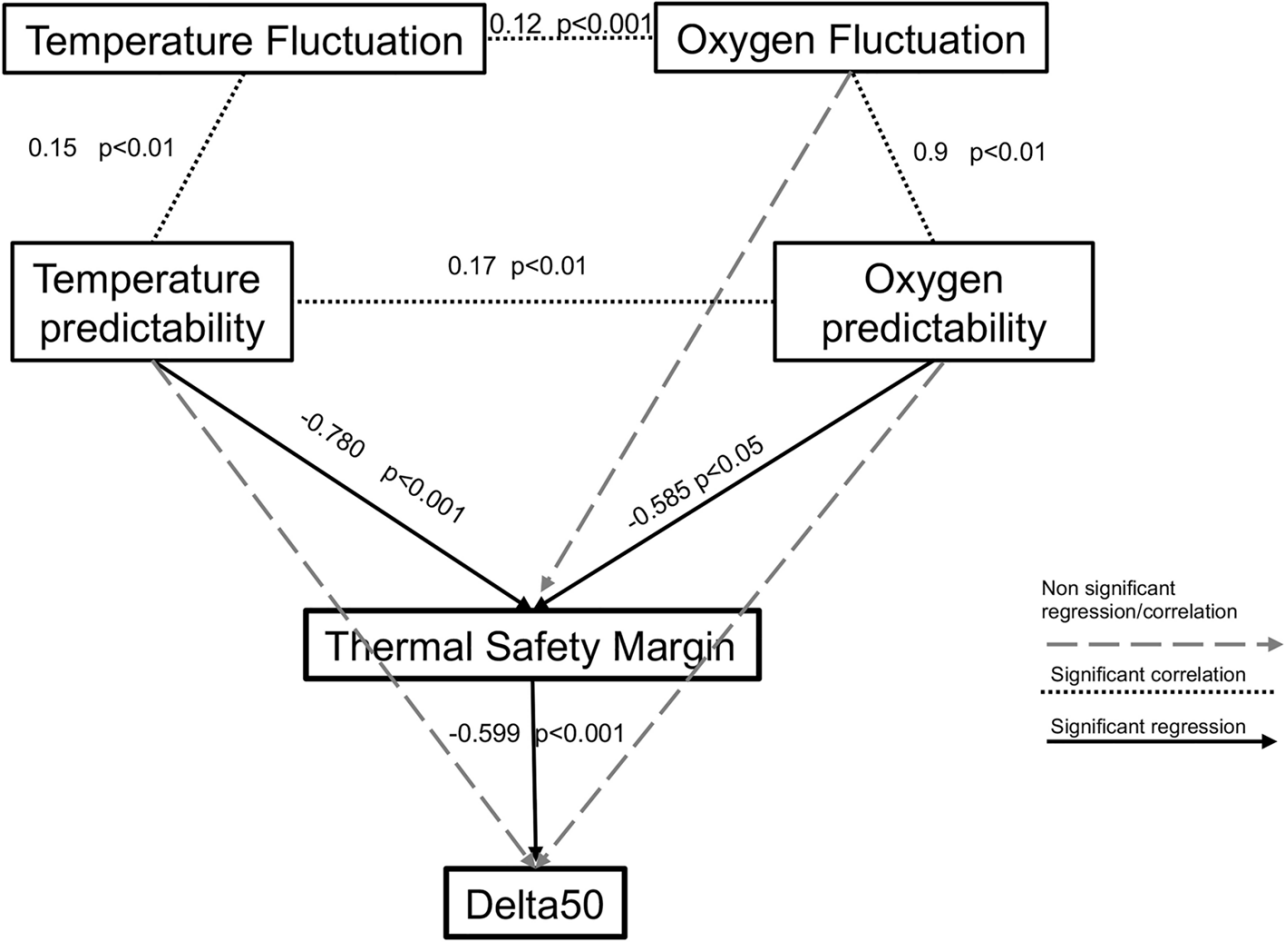
Final path model describing how the compensation of the thermal response under ecologically relevant conditions (i.e., oxygen level, ΔLT50) is shaped by the Thermal Safety Margins that in turn are controlled by the fluctuations and predictability of oxygen and temperature. Temperature and oxygen fluctuations are correlated among themselves and with temperature predictability and oxygen predictability, respectively. Temperature and Oxygen predictability are significantly correlated. Thermal safety margins are significantly and negatively determined by the predictability of oxygen and temperature which means that lower predictability is related to higher safety margins, principally because species living in colder environments have higher thermal safety margins and lower oxygen and temperature predictability. In turn, thermal safety margins inversely affect the species ΔLT50. This means that higher thermal safety margins determine lower ΔLT50 in the thermal response when exposed to supersaturation and lower thermal safety margin determines a higher ΔLT50 when the species is exposed to supersaturation. Path diagram with standardized path coefficients reported next to the black solid arrows with a significant p value (p < 0.05). Significant positive correlations are reported with dotted lines. Non-significant pathways are given in dashed grey shafts.

The final path model (Fig. 4; Supplementary table S5; Comparative Fit Index (CFI) 0.902 and RMSEA 0.09) revealed a significant correlation among oxygen and temperature fluctuations and their predictability, while the predictability of these two parameters had an inverse effect on the thermal safety margins (higher predictability means lower safety margins for the species). The thermal safety margin, in turn, is inversely correlated with the gain in thermal tolerance under conditions of supersaturation, showing that the lower the thermal safety margin, the higher the capability of the species to display compensatory mechanisms under ecologically relevant environmental oxygen saturation.

## Discussion

In marine habitats, environmental variability occurs over multiple temporal scales, including tidal and diurnal cycles, seasonal variations and occasional stochastic events^19,20^. The different periodicity, frequency of occurrence and magnitude of environmental variability affects the nature and scale of the physiological responses of marine organisms. The present data showed that the three productive coastal marine ecosystems have different regimes of oxygen and temperature fluctuations, particularly at the daily scale where the dominant oscillations are found. While the Tropical site exhibits a consistently wide range of high temperature and oxygen (up to 200% of saturation) fluctuations, the Warm Temperate site shows a clear seasonality with low temperatures associated with low oxygen variation in the winter and higher temperatures associated with higher oxygen variation during the summer, principally driven by the enhanced photosynthetic activity of primary producers. The summer rate of oxygen variation reaches values similar to that of the Tropical one. Notably, the Warm Temperate site has the largest thermal variation, ranging from almost 0 in winter to up to 40 °C in summer, compared to the other two sites, which are constrained within a narrower range of thermal variability. At the Cold Temperate site, the lowest range of temperature and oxygen variation was recorded with only marginal supersaturation during winter (up to 140%) and mild oxygen undersaturation (~ 60%). This evidences the thermal constraints on the primary producers found in cold coastal waters like the North Sea^21^. Temperature is a well-known limiting factor for the growth of aquatic primary producers^22^. Furthermore, low temperature depresses the rate of photosynthesis in many seaweed and cyanobacteria species, constraining the activity of photosynthetic enzymes involved in the Calvin cycle, such as Rubisco^23,24^, limiting primary production^25,26^.

There is now a growing consensus on the need to investigate the physiological responses and phenotypic plasticity of organisms living in routinely fluctuating environments^11,27^, particularly in connection to environmental predictability^1,28^. The regimes of dissolved oxygen and water temperature fluctuations in the sites investigated here are characterized by distinct levels of predictability, peaking at more than 90% at tropical latitudes, while at temperate latitudes they decrease to 71% in the Cold Temperate site and to 62% in the Warm Temperate site (Table 1). This difference has important implications for the mechanism of the physiological response of organisms inhabiting the different sites because they are routinely subjected to latitude-specific differences in the regularity of environmental variation. The latitudinal diversity in environmental predictability (i.e., highest values in the tropics, lowest in the temperate regions) in productive coastal waters observed here, is congruent with findings of atmospheric circulation, recently modelled at planetary scales^29,30^. Specifically, the predictability of the atmosphere, which has pivotal implications for weather and associated environmental variation, is much higher over the longer term at tropical compared to extratropical latitudes. This is due to the equatorial waves being significantly more consistent than middle-latitude baroclinic disturbances, allowing a higher weather predictability at low latitudes^29,30^.

The synchrony of oxygen and temperature fluctuations is an important feature of productive aquatic habitats and has recently been described for tropical^6^, temperate^8,31^, polar environments^32^ and even freshwater habitats^33,34^. The influence of the predictability of environmental fluctuations on the plasticity of physiological response of aquatic organisms has been investigated with promising results. Bitter et al.^35^ showed that predictable pH fluctuations enhance the metabolic response of the Mediterranean mussel, *Mytilus galloprovincialis*, while a decrease in pH predictability depresses phenotypic plasticity in natural populations. This provides empirical support to recent theoretical studies^36,37^ that have demonstrated how environmental predictability and the timescale of environmental variations shape the plastic phenotypic response of the mussels.

The present data from 17 marine species belonging to 6 different phyla showed a significant effect of the predictability of oxygen and temperature fluctuations on the plasticity of the animals’ heat tolerances when exposed to oxygen supersaturation. In accordance with other recent findings^6–8,12,38^, the oxygen supersaturation occurring at the three sites investigated here significantly increased the heat tolerance of the studied species. Indeed, oxygen supersaturation can fulfil increased metabolic demand during heating events in the environment^6,12^. However, the magnitude of the effect of oxygen supersaturation differed according to the latitude studied. Tropical animals had the largest increase in heat tolerance when exposed to supersaturation, while animals from the warm temperate latitudes had the lowest increase. We interpreted these results considering that species living in constant higher temperatures (i.e., in the tropics) are thermal specialists compared to thermal generalists that have evolved under colder and more stochastic temperature regimes^39,40^. Thermal generalists can sustain their metabolic rate across a wide thermal niche due to compensatory mechanisms of respiratory functions^41^ which can allow them to perform well under irregularly varying environmental conditions. In this study, species living at temperate latitudes had a narrower heat response to oxygen fluctuations compared to those living at the tropics, likely resulting from large compensatory mechanisms expressed in the large range of temperatures they experience^42,43^. In contrast, the tropical thermal specialists, confined to a narrower thermal niche, revealed a broad phenotypic plasticity, which markedly extended their thermal tolerance when exposed to supersaturation. These results provide empirical validation for the theoretical model developed by Botero et al.^36^ predicting that rather than the amplitude of environmental oscillations it is the predictability of the environment which drives the phenotypic plasticity of organisms to multiple and novel environmental stressors^9,44^.

Predictable fluctuations of pH and oxygen in aquatic environments significantly expand the physiological tolerances of aquatic organisms^8,27,34^. These results show that oxygen and temperature predictability allow animals to widen the gain in heat tolerance driven by oxygen supersaturation, at least in the species studied. Furthermore, these results demonstrate that environmental predictability not only drives the thermal response of the marine species, but significantly extends the heat tolerance of thermal specialists when exposed to oxygen supersaturation thus broadening their thermal safety margin. The organisms from tropical latitudes, that evolved under high environmental predictability, are adapted to anticipate daily cyclical change in environmental conditions, which allows them to allocate energy more efficiently in order to endure novel conditions despite the magnitude of the change experienced^1,45^, in accordance with the feedforward phenotypic plasticity of physiological response. Organisms living at high latitudes are less efficient in exploiting the protective effects of oxygen supersaturation but display broader thermal safety margins, since they are exposed to more stochastic diel oxygen and temperature variations due to climatic and weather conditions, and consequently to lower environmental predictability. For this reason, species at high latitudes are more prone to rely on feedback phenotypic plasticity enduring environmental cues with compensatory mechanisms (i.e., metabolic depression and/or expression of heat shock proteins) rather than adopting feedforward phenotypic plasticity driven by environmental predictability^36^. When environmental conditions fluctuate constantly, and are thus, highly predictable, individuals can tune their phenotype to cope with prevailing conditions at diel and seasonal scale and likely also with novel climate events^9^. In the case of environments characterised by unpredictable variability, like in high latitudes, acclimatisation can come at a high cost. Failure to anticipate future conditions due to stochastic environmental variability plays a decisive role in shaping the thermal tolerance of species because it results in a costly subsequent change of the organisms’ thermal response^2,34,35,46–48^. Such a bet-hedging strategy is promoted in highly stochastic environments because it allows organisms to persist in stressful conditions but at a high cost in terms of fitness^36,49^.

## Conclusion

Our study suggests that differences in environmental predictability can play a role in driving different physiological responses in marine species. Specifically, species exposed to predictable environments can tune their physiology accordingly. In contrast, species in non-predictable environments face stochastic events that increase the diverse bet-hedging strategies allowing them to cope with stressful conditions^36^.

Both the thermal safety margins of marine organisms and the predictability of the environmental fluctuations they are exposed to are affected by climate change, since the latter may decouple the correlations between environmental variables. To allow better forecasting of organism vulnerability and ecosystem resilience under climate change, we advocate the integration of environmental fluctuations and their predictability into future studies.

## Material and methods

### Study sites

Our study sites were located in the central Red Sea, in a coastal area characterised by fringing mangroves at the “Ibn Sina” field research station (22°20′19.90″N, 39°5′24.30″E) of the King Abdullah University for Science and Technology (KAUST, Saudi Arabia), in the Venice lagoon (45°13′37.66″N, 12°16′10.48″E) nearby the City of Chioggia (Italy) in an area characterized by the presence of marine macrophyte beds and seagrass meadows dominated by the common eelgrass (*Zostera marina*), and in Scotland (UK) in the rocky shoreline (55°53′53.20″N, 2° 7′41.83″W) at the St. Abbs Marine Station, characterized by the presence of massive bladder wrack algae (*Fucus vesiculosus*) and other marine macrophytes. The facilities used to run the experiments were, respectively, the ESM Lab at KAUST, the Chioggia Hydrobiological Station “Umberto D’Ancona” of the University of Padova (chioggia.biologia.unipd.it/en), and the St. Abbs Marine Station (marinestation.co.uk). The three sites are characterized by different climates and based on the Köppen-Geiger classification (http://koeppen-geiger.vu-wien.ac.at/present.htm) Saudi Arabia was considered Arid and hereafter referred to as the Tropical Site, Venice Lagoon, considered a Mediterranean climate, and hereafter referred to as the Warm Temperate site, and St. Abbs (Scotland), considered Oceanic climate, and hereafter referred as the Cold Temperate site. In all locations the sampling was carried out within 5 m depth.

### Species investigated

To characterize the effect of oxygen supersaturation in enhancing the heat tolerance of marine ectotherms, we sampled six different phyla in the study sites considered. For the Tropical site, we retrieved published data^6^ for six species. Specifically, 14 individuals of *Thalamita crenata* (Arthropoda: Portunidae), 77 of *Atherinomorus *sp. (Chordata: Atherinidae), 28 of *Holothuria atra* (Echinodermata: Holothuroidae), 58 of *Dascyllus *sp. (Chordata: Pomacentridae), 46 of *Ophiocoma *sp. (Echinodermata: Ophiocomidae) and 46 individuals of *Modiolus *sp. (Mollusca: Mytilidae).

For the Warm Temperate site, in July 2018 we collected 36 individuals of *Caprella *sp. (Arthropoda: Caprellidae), 42 of *Gammarus *sp. (Arthropoda: Gammaridae), 42 of *Haminoea *sp. (Mollusca: Haminoeidae), 42 of *Paracentrotus *sp. (Echinodermata: Parechinidae), 42 of *Ophitrix *sp. (Echinodermata: Ophiocomidae) and 42 individuals of *Mytilus *sp. (Mollusca: Mytilidae).

For the Cold Temperate site, in August 2018 we collected 12 individuals of *Necora *sp. (Arthropoda: Portunidae), 20 of *Ostrea *sp. (Mollusca: Ostreidae), 20 of *Echinus *sp. (Echinodermata: Echinidae), 12 of *Ophitrix *sp. (Echinodermata: Ophiocomidae) and 16 individuals of *Mytilus *sp. (Mollusca: Mytilidae).

This project was completed under the Saudi Arabia and KAUST ethics permit (Institutional Animal Care and Use Committee approval 18IACUC10) and all experiments were performed in accordance with relevant guidelines and regulations complying with ARRIVE guidelines (https://arriveguidelines.org) and the necessary requirement by the three countries where the experiments were performed.

### Environmental data logging

To retrieve dissolved oxygen concentration and water temperature time series from the study sites we deployed three PME miniDOT^®^ Dissolved Oxygen Loggers from August 2016 to August 2017 and February 2017 to July 2018 for Saudi Arabia, from April 2017 to May 2018 for Venice lagoon and August 2017 to July 2018 for St. Abbs Marine station. The loggers were positioned in shallow water environments at the transition between the benthic boundary layer and the open water, in close proximity to the primary producers to better assess the influence of in situ metabolic activity on environmental parameters (following^4,6^).

Observations of dissolved oxygen concentration and corresponding water temperature were available for the three habitats every 10 min, during day and night. Dissolved oxygen concentration at 100% saturation in seawater was calculated from the PME miniDOT^®^.

Loggers were periodically cleaned to avoid fouling interference with oxygen and temperature measure and data quality was checked: all the measured values reported a data quality threshold above 98% recorded from the logger, indicating the robustness of the data.

Before further analysis, the time series of oxygen and temperature were detrended using the R package *astsa*^50^. The daily oxygen and temperature variability was calculated by subtracting the daily minimum to the daily maximum value of temperature and oxygen saturation along the logging period. The relationship between these two new variables was tested considering the temperature range as explanatory variable and the oxygen range as response variable.

### Mortality rate LT50

Temperature-induced mortality rates and LT50s were measured following the method described by Giomi and colleagues^51^. For each species, two groups of individuals underwent different treatments: half of the animals were exposed to a level of dissolved oxygen saturation of 97 ± 2% (full saturation), and half to oxygen supersaturation around 140 ± 3% for 2 h before the start of temperature ramping. These two levels of oxygen saturation were comparable to those observed in the habitats examined (Fig. 1). Temperature ramping was set according with the natural oscillation experienced by the animal at the site, thereby mimicking the site-specific environmental temperature fluctuations. Specifically, the ramp was set at 1 °C every 30 min for animals in the Tropical and Warm Temperate sites, and 0.5 °C every 60 min for the Cold Temperate site. The temperature ranges tested were 25–49 °C for the Tropical site, 26–46 °C for the Warm Temperate and 15.5–37.5 °C for the Cold Temperate. Dissolved oxygen concentration was monitored during the entire experiment using a calibrated multiparametric probe (SevenGo Duo Pro SG98 Portable RDO/pH/Ion Meter, Mettler Toledo Instruments) and adjusted to maintain supersaturation by regulating the flow of air and nitrox (O_2_ at 36%). The mortality of individuals (defined through the nonreactivity to tactile stimulus) was assessed every 30 min. Dead individuals were immediately removed from the experimental tank, and the experiments were terminated when all animals were dead.

Prior to the experiment, the animals collected were transferred into the aquarium facilities and allowed to recover from handling stress for 24 h in saturated (97% of oxygen saturation) filtered seawater, according to the procedures described by Fusi and colleagues^40^, without feeding. In the laboratory, the acclimation temperature was set to mimic the field temperature during the night-time when animals were collected.

### Wavelet and cross-wavelet analysis

Wavelet analysis was used to identify the dominant periodicities in the dissolved oxygen or temperature time series and depict whether they are taking place as regular, periodic oscillations or they are only found in certain moments or periods, for example as isolated or intermittent bursts. To compute the continuous wavelet power spectrum^52^ of the temperature and dissolved oxygen time series, each time series was percentile transformed and analysed using the Morlet wavelet and the significance levels were assessed against a null hypothesis of red noise. The wavelet power spectrum describes, for each moment of the time span covered by the time series, the distribution of the variability in the time series as a function of different frequencies (or periods) of oscillation. This analysis allowed us to determine the relative contribution of each period (or frequency) of oscillation to the total variation observed in each temperature and oxygen time series, as well as how this contribution is spread over the time span covered by the time series. The analysis was limited to the longest period of each time series without gaps (except for minor gaps which were interpolated (e.g. in the case of a few missing hours in a day), or filled with observations from nearby days, as appropriate), as required by the statistical methodology. Calculations were performed using Grinsted’s MATLAB toolbox which can be found at https://noc.ac.uk/business/marine-data-products/cross-wavelet-wavelet-coherence-toolbox-matlab.

We also performed a cross wavelet analysis^52^ for the temperature and oxygen time series in each location. This analysis allowed the investigation of whether the two-time series co-vary and, if so, if they co-vary only in the case of certain frequencies of oscillation and if there is a consistent phase relationship between their oscillations.

To compute the typical amplitude of the daily fluctuations in water temperature or dissolved oxygen concentration over the monitored period, we carried out a Fast Fourier Transform (FFT) analysis of each time series and extracted the amplitude corresponding to a 24 h frequency from the resulting amplitude spectrum.

### Predictability

The amount of predictability of the temperature or oxygen fluctuations was computed with reference to a periodicity of 24 h, i.e., the dominant period identified in the time series by the wavelet analysis. The predictability of a time series was defined as the fraction of the monitored time frame during which a statistically significant (as identified by the wavelet analysis) 24 h periodicity was present for that time series. To calculate the duration of the monitored time frame, only the period not covered by the cone of influence corresponding to the 24 h periodicity was considered, because wavelet outputs cannot be reliably interpreted within the cone of influence, i.e., close to the start and end of the time series^52^.

### Statistical analyses

Differences in LT50 were assessed using the R package *survival*^53^ and the graphs were prepared using the R package *drc*^54^.

Thermal safety margins in Fig. 3 were calculated following Sunday and colleagues^21^; briefly, we computed the thermal safety margins by calculating the difference between each species LT50 when exposed to supersaturation (140%) and the mean of the daily maximum temperatures experienced by that species for each site (Fig. 3A). For comparison, they were also calculated as the difference between each species LT50 when exposed to full saturation (97%) and the mean of the maximum temperature experienced by that species for each site (Fig. 3B). We used a linear model to test the relationship between the safety margins and ΔLT50, calculated as the difference between the LT50 of the animal exposed to supersaturation and full saturation.

To quantify relationships between the oxygen and temperature fluctuation, predictability, thermal safety margins, and the ΔLT50 defined as the difference between LT50 measured in supersaturation and full saturation of oxygen, we performed a path analysis using the package *lavaan*^55^ in R. This confirmatory path analysis method enabled the overall fit of a multifaceted network of causality to be tested, including the estimation of indirect effects, while allowing the use of a nested data structure. To derive comparable estimates, all quantitative predictors (i.e., temperature and oxygen), thermal safety margins and ΔLT50 were standardized to a mean of zero and standard deviation of one. Based on the literature (i.e.,^6^) we constructed an a priori model with hypothesized pathways among oxygen and temperature fluctuations, predictability (all exogenous variables, i.e., variables whose variation is a result of components outside the model), thermal safety margins, and the ΔLT50 of the species. We used a multiple hypothesis testing to select the best model to describe the relationship among our variables and the best model was accepted based on the Fisher’s C statistic and the best Comparative Fit Index (CFI) and RMSEA.

## Supplementary Information

Supplementary Information 1.

Supplementary Information 2.

## Data Availability

All the data are provided as Supplementary tables data 1 (Table S1A–D, S2–5) to this manuscript and the scripts used for analysing those data are available at GitHub https://github.com/MarcoFusi1980/Oxygen_Variability_and_Predicatibility_01.

## References

[1] Bernhardt, J. R., O’Connor, M. I., Sunday, J. M. & Gonzalez, A. Life in fluctuating environments. *Philos. Trans. R. Soc. Lond. B Biol. Sci.* **375**, 20190454 (2020). 33131443 10.1098/rstb.2019.0454 PMC7662201

[2] Kroeker, K. J. et al. Ecological change in dynamic environments: Accounting for temporal environmental variability in studies of ocean change biology. *Glob. Change Biol.* **26**, 54–67 (2020). 10.1111/gcb.14868 31743515

[3] Helmuth, B. et al. Mosaic patterns of thermal stress in the rocky intertidal zone: Implications for climate change. *Ecol. Monogr.* **76**, 461–479 (2006).

[4] Giomi, F. et al. Oxygen dynamics in marine productive ecosystems at ecologically relevant scales. *Nat. Geosci.* **16**, 560–566 (2023).

[5] Lima, F. P. & Wethey, D. S. Three decades of high-resolution coastal sea surface temperatures reveal more than warming. *Nat. Commun.* **3** (2012). 10.1038/ncomms1713 22426225

[6] Giomi, F. et al. Oxygen supersaturation protects coastal marine fauna from ocean warming. *Sci. Adv.* **5**, 1–8 (2019). 10.1126/sciadv.aax1814 PMC6726443 31517051

[7] Fusi, M., Daffonchio, D., Booth, J. & Giomi, F. Dissolved oxygen in heterogeneous environments dictates the metabolic rate and thermal sensitivity of a tropical aquatic crab. *Front. Mar. Sci.* **8**, 1–9 (2021).35685121

[8] Booth, J. et al. Diel oxygen fluctuation drives the thermal response and metabolic performance of coastal marine ectotherms. *Proc. R. Soc. B Biol. Sci.* **288**, 20211141 (2021). 10.1098/rspb.2021.1141 PMC8220261 34157869

[9] Bitter, M. C. *et al.* Fluctuating selection and global change: A synthesis and review on disentangling the roles of climate amplitude, predictability and novelty. *Proc. R. Soc. B Biol. Sci.* **288** (2021). 10.1098/rspb.2021.0727 PMC8385344 34428970

[10] Bitter, M. C. *et al.* The importance of incorporating natural thermal variation when evaluating physiological performance in wild species. *J. Exp. Biol.* **221** (2018). 10.1242/jeb.164673 30037965

[11] Cabrerizo, M. J. & Marañón, E. Net effect of environmental fluctuations in multiple global-change drivers across the tree of life. *Proc. Natl. Acad. Sci. USA* **119**, 1–8 (2022). 10.1073/pnas.2205495119 PMC9371701 35914141

[12] McArley, T. J., Morgenroth, D., Zena, L. A., Ekström, A. T. & Sandblom, E. Prevalence and mechanisms of environmental hyperoxia-induced thermal tolerance in fishes. *Proc. R. Soc. B Biol. Sci.* **289** (2022). 10.1098/rspb.2022.0840 PMC9382203 35975439

[13] Harada, A. E. & Burton, R. S. Ecologically relevant temperature ramping rates enhance the protective heat shock response in an intertidal ectotherm. *Physiol. Biochem. Zool.* **92**, 152–162 (2019). 30694107 10.1086/702339

[14] Cleves, P. A. et al. Reduced thermal tolerance in a coral carrying CRISPR-induced mutations in the gene for a heat-shock transcription factor. *Proc. Natl. Acad. Sci. USA* **117**, 28899–28905 (2020). 33168726 10.1073/pnas.1920779117 PMC7682433

[15] Vannini, M., Lori, E., Coffa, C. & Fratini, S. Cerithidea decollata: a snail that can foresee the future?. *Anim. Behav.* **76**, 983–992 (2008).

[16] Bennett, J. M. et al. GlobTherm, a global database on thermal tolerances for aquatic and terrestrial organisms. *Sci. Data* **5**, 1–7 (2018). 29533392 10.1038/sdata.2018.22 PMC5848787

[17] Pinsky, M. L., Eikeset, A. M., McCauley, D. J., Payne, J. L. & Sunday, J. M. Greater vulnerability to warming of marine versus terrestrial ectotherms. *Nature* **569**, 108–111 (2019). 31019302 10.1038/s41586-019-1132-4

[18] Sunday, J. M. et al. Thermal-safety margins and the necessity of thermoregulatory behavior across latitude and elevation. *Proc. Natl. Acad. Sci. USA* **111**, 5610–5615 (2014). 24616528 10.1073/pnas.1316145111 PMC3992687

[19] Blauw, A. N., Benincà, E., Laane, R. W. P. M., Greenwood, N. & Huisman, J. Predictability and environmental drivers of chlorophyll fluctuations vary across different time scales and regions of the North Sea. *Prog. Oceanogr.* **161**, 1–18 (2018).

[20] Guadayol, Ò., Silbiger, N. J., Donahue, M. J. & Thomas, F. I. M. Patterns in temporal variability of temperature, oxygen and pH along an environmental gradient in a coral reef. *PLoS One* **9** (2014). 10.1371/journal.pone.0085213 PMC3885695 24416364

[21] Serrão, João Neiva, E. A. et al. *Seaweed Phylogeography: Adaptation and Evolution of Seaweeds under Environmental Change*. *Seaweed Phylogeography*. 10.1007/978-94-017-7534-2 (2016).

[22] Jørgensen, S. & Bendoricchio, G. *Fundamentals of Ecological Modelling* (Elsevier, 2021).

[23] Yamori, W., Noguchi, K., Hikosaka, K. & Terashima, I. Phenotypic plasticity in photosynthetic temperature acclimation among crop species with different cold tolerances. *Plant Physiol.* **152**, 388–399 (2010). 19880611 10.1104/pp.109.145862 PMC2799372

[24] Iñiguez, C., Galmés, J. & Gordillo, F. J. L. Rubisco carboxylation kinetics and inorganic carbon utilization in polar versus cold-temperate seaweeds. *J. Exp. Bot.* **70**, 1283–1297 (2019). 30576461 10.1093/jxb/ery443 PMC6382342

[25] Davey, M. C. The effects of freezing and desiccation on photosynthesis and survival of terrestrial Antarctic algae and cyanobacteria. *Polar Biol.* **10**, 29–36 (1989).

[26] Smith, C. M. & Berry, J. A. Oecologia to osmotic and temperature stresses: comparative studies of species with differing distributional limits. *Response* 6–12 (1986). 10.1007/BF00377105 28311281

[27] Vargas, C. A. et al. Upper environmental pCO2 drives sensitivity to ocean acidification in marine invertebrates. *Nat. Clim. Change* **12**, 200–207 (2022).

[28] Jiang, M., Felzer, B. S., Nielsen, U. N. & Medlyn, B. E. Biome-specific climatic space defined by temperature and precipitation predictability. *Glob. Ecol. Biogeogr.* **26**, 1270–1282 (2017).

[29] Straus, D. M. & Paolino, D. Intermediate time error growth and predictability: Tropics versus mid-latitudes. *Tellus Ser. A Dyn. Meteorol. Oceanogr.* **61**, 579–586 (2009).

[30] Judt, F. Atmospheric predictability of the tropics, middle latitudes, and polar regions explored through global storm-resolving simulations. *J. Atmos. Sci.* **77**, 257–276 (2020).

[31] McArley, T. J., Hickey, A. J. R. & Herbert, N. A. Hyperoxia increases maximum oxygen consumption and aerobic scope of intertidal fish facing acutely high temperatures. *J. Exp. Biol.* **221** (2018). 10.1242/jeb.189993 30254026

[32] Krause-Jensen, D. et al. Long photoperiods sustain high pH in Arctic kelp forests. *Sci. Adv.* **2**, e1501938 (2016). 27990490 10.1126/sciadv.1501938 PMC5156516

[33] Andersen, M. R., Kragh, T. & Sand-Jensen, K. Extreme diel dissolved oxygen and carbon cycles in shallow vegetated lakes. *Proc. Biol. Sci.* **284**, 20171427 (2017). 28904141 10.1098/rspb.2017.1427 PMC5597838

[34] Booth, J. M., Giomi, F., Daffonchio, D., Mcquaid, C. D. & Fusi, M. Disturbance of primary producer communities disrupts the thermal limits of the associated aquatic fauna. *Sci. Total Environ.* **872**, 162135 (2023). 36775146 10.1016/j.scitotenv.2023.162135

[35] Bitter, M. C., Kapsenberg, L., Silliman, K., Gattuso, J. P. & Pfister, C. A. Magnitude and predictability of pH fluctuations shape plastic responses to ocean acidification. *Am. Nat.* **197**, 486–501 (2021). 33755541 10.1086/712930

[36] Botero, C. A., Weissing, F. J., Wright, J. & Rubenstein, D. R. Evolutionary tipping points in the capacity to adapt to environmental change. *Proc. Natl. Acad. Sci. USA* **112**, 184–189 (2015). 25422451 10.1073/pnas.1408589111 PMC4291647

[37] Bonamour, S., Chevin, L. M., Charmantier, A. & Teplitsky, C. Phenotypic plasticity in response to climate change: The importance of cue variation. *Philos. Trans. R. Soc. B Biol. Sci.* **374** (2019). 10.1098/rstb.2018.0178 PMC6365871 30966957

[38] McArley, T. J., Morgenroth, D., Zena, L. A., Ekström, A. T. & Sandblom, E. Experimental hyperoxia (O2 supersaturation) reveals a gill diffusion limitation of maximum aerobic performance in fish. *Biol. Lett.* **18**, 20220401 (2022). 36321431 10.1098/rsbl.2022.0401 PMC9627442

[39] Verberk, W. C. E. P. *et al.* Can respiratory physiology predict thermal niches? *Ann. N. Y. Acad. Sci.* 1–16. 10.1111/nyas.12876 (2015). 10.1111/nyas.12876 26333058

[40] Fusi, M. et al. Thermal specialization across large geographical scales predicts the resilience of mangrove crab populations to global warming. *Oikos* **124**, 784–795 (2015).

[41] Huey, R. B. & Kingsolver, J. G. Climate warming, resource availability, and the metabolic meltdown of ectotherms. *Am. Nat.* **194**, E140–E150 (2019). 31738103 10.1086/705679

[42] Reusch, T. B. H. Climate change in the oceans: Evolutionary versus phenotypically plastic responses of marine animals and plants. *Evol. Appl.* **7**, 104–122 (2014). 24454551 10.1111/eva.12109 PMC3894901

[43] Broitman, B. R., Aguilera, M. A., Lagos, N. A. & Lardies, M. A. Phenotypic plasticity at the edge: Contrasting population-level responses at the overlap of the leading and rear edges of the geographical distribution of two Scurria limpets. *J. Biogeogr.* **45**, 2314–2325 (2018).

[44] Bujan, J., Roeder, K. A., Yanoviak, S. P. & Kaspari, M. Seasonal plasticity of thermal tolerance in ants. *Ecology* **101**, 1–6 (2020). 10.1002/ecy.3051 32239508

[45] Sokolova, I. M., Frederich, M., Bagwe, R., Lannig, G. & Sukhotin, A. A. Energy homeostasis as an integrative tool for assessing limits of environmental stress tolerance in aquatic invertebrates. *Mar. Environ. Res.* **79**, 1–15 (2012). 22622075 10.1016/j.marenvres.2012.04.003

[46] Angilletta, M. J. Jr. & Angilletta, M. J. *Thermal Adaptation: A Theoretical and Empirical Synthesis* (Oxford University Press, 2009).

[47] Angilletta, M. J., Cooper, B. S., Schuler, M. S. & Boyles, J. G. The evolution of thermal physiology in endotherms. *J. Therm. Biol.* **2**, 249–268 (2002). 10.2741/e148 20515760

[48] Angilletta, M. J., Niewiarowski, P. H. & Navas, C. A. The evolution of thermal physiology in ectotherms. *J. Therm. Biol.* **27**, 249–268 (2002).

[49] Morawska, L. P., Hernandez-Valdes, J. A. & Kuipers, O. P. Diversity of bet-hedging strategies in microbial communities—Recent cases and insights. *WIREs Mech. Dis.* **14**, 1–15 (2022). 10.1002/wsbm.1544 PMC9286555 35266649

[50] Shumway, R. H. & Stoffer, D. S. *Time Series: A Data Analysis Approach Using R*. (Chapman and Hall/CRC, 2019). 10.1201/9780429273285

[51] Giomi, F. et al. The importance of thermal history: Costs and benefits of heat exposure in a tropical, rocky shore oyster. *J. Exp. Biol.* **219**, 686–694 (2016). 26747904 10.1242/jeb.128892

[52] Grinsted, A., Moore, J. C. & Jevrejeva, S. Application of the cross wavelet transform and wavelet coherence to geophysical time series. *Nonlinear Process Geophys.* **11**, 515–533 (2004).

[53] Moore, D. F. *Applied Survival Analysis Using R* (Springer International Publishing, Cham, 2016). 10.1007/978-3-319-31245-3

[54] Knezevic, S. Z., Streibig, J. C. & Ritz, C. Utilizing R software package for dose-response studies: the concept and data analysis. *Weed Technol.* **21**, 840–848 (2007).

[55] Rosseel, Y. lavaan: An R package for structural equation modeling. *J. Stat. Softw.* **48** (2012).

