# Supplementary material for: The predictability of fluctuating environments shapes the thermal tolerance of marine ectotherms and compensates narrow safety margins
Source: Sci Rep. 2024 Oct 30;14:26174. doi: 10.1038/s41598-024-77621-1 (PMC11526141; doi:10.1038/s41598-024-77621-1)
Supplement: Supplementary file 1 — Supplementary Information 1. [file 41598_2024_77621_MOESM1_ESM.pdf]

Supplementary Material

The predictability of fluctuating environments shapes the thermal tolerance of marine ectotherms and compensates for narrow safety margins

Authors:

Marco Fusi1,2,3,*†, Alberto Barausse4,5,6 Jenny Marie Booth7,8 Erica Chapman9, Daniele Daffonchio2, William Sanderson10, Karen Diele3,11, Folco Giomi12*†,

Affiliation:

1 Dove Marine Laboratory, School of Natural and Environmental Sciences, Newcastle University, Newcastle NE1 7RU, UK

2 King Abdullah University of Science and Technology (KAUST), Red Sea Research Center (RSRC), Thuwal 23955-6900, Saudi Arabia

3 Centre for Conservation and Restoration Science, Edinburgh Napier University, Sighthill Campus, Edinburgh, UK

4 Department of Biology, University of Padua, via U. Bassi 58/b, 35131 Padova, Italy.

5 Department of Industrial Engineering, University of Padova, via Gradenigo 6/a, 35131 Padova, Italy

6 National Biodiversity Future Center (NBFC), Piazza Marina 61, 90133, Palermo, Italy

7 Joint Nature Conservation Committee, Monkstone House, City Road, Peterborough, PE1 1JY, UK

8 Coastal Research Group, Department of Zoology and Entomology, Rhodes University, PO Box 94, Grahamstown 6140, South Africa

9 St Abbs Marine Station, St Abbs, UK

10 Centre for Marine Biodiversity & Biotechnology, ILES, EGIS, Heriot-Watt University, Edinburgh, UK

11 School of Applied Science, Edinburgh Napier University, Sighthill Campus, Edinburgh, UK

12 Department of Biology, University of Rome Tor Vergata, Rome, Italy

*Corresponding authors. Email: marco.fusi@newcastle.ac.uk (M.F.); folcog@gmail.com (F.G.)

†These authors contributed equally to this work.

Supplementary Figures

Supplementary figure S1. Dissolved oxygen concentration and water temperature in the three locations, Cold, Warm Temperate and Tropical between 2017 and 2018. Oxygen concentration is plotted for 1°C temperature intervals; the violin plots represent the distribution of the observations; the red line indicates oxygen concentration at the water saturation (100% of saturation). The full oxygen frequency distribution data are represented in the Kernel plots on the left side.

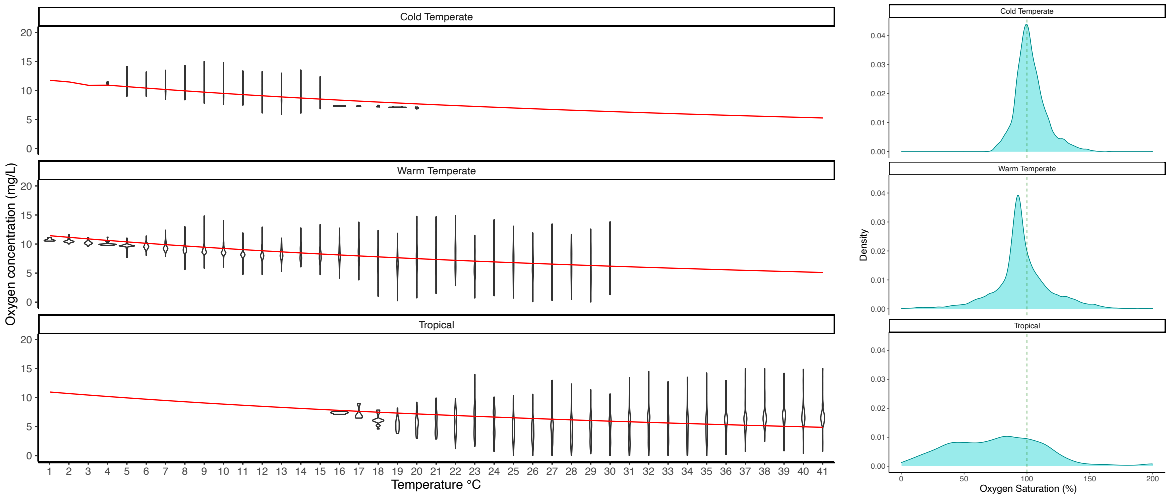

Supplementary figure S2. Thermal tolerance of 17 marine species (6 phyla) measured as lethal temperatures (LT50) for 50% of the individuals exposed to full oxygen saturation (red line) and supersaturation (cyan line).

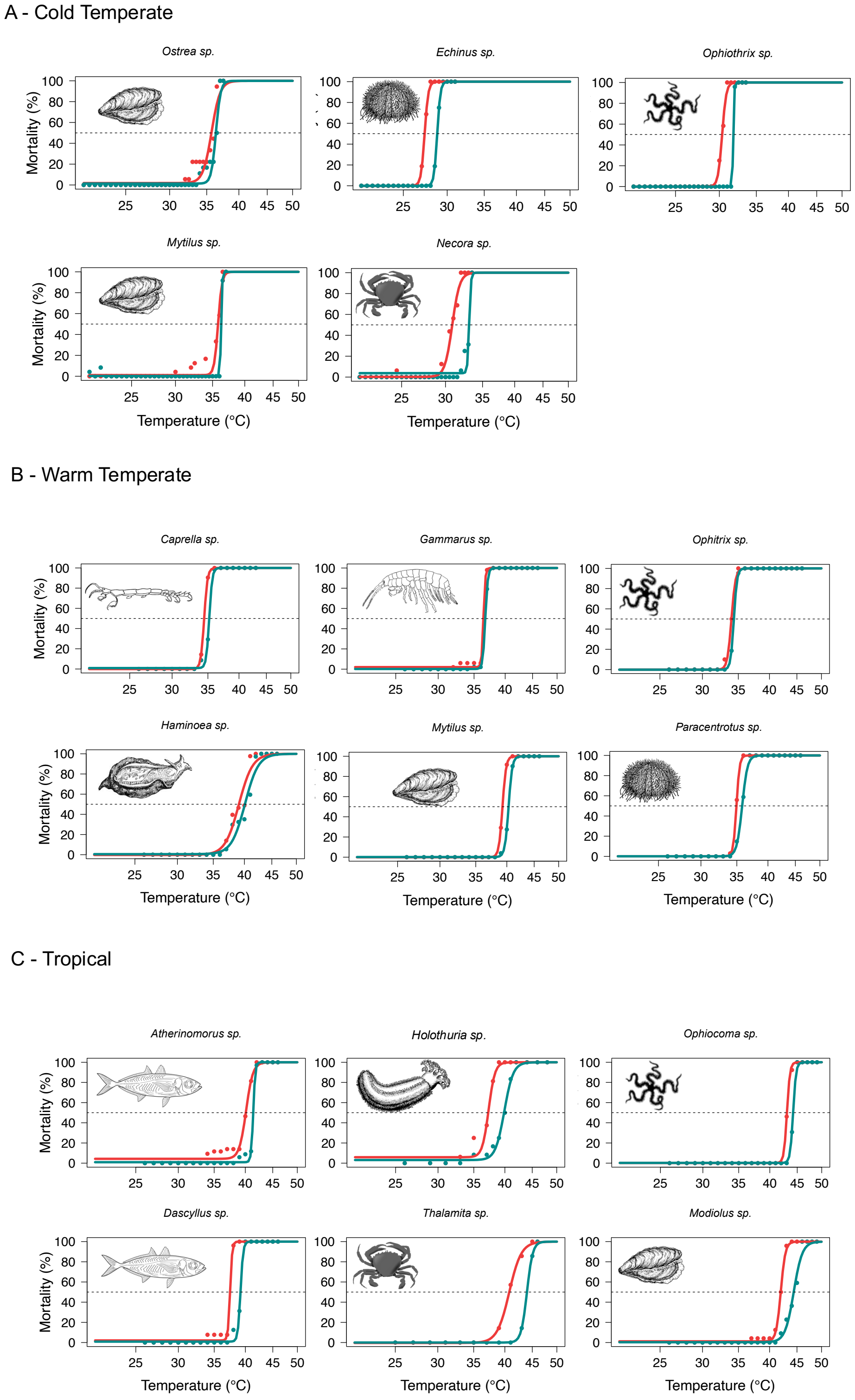
A - Cold Temperate
Ostrea sp.
Echinus sp.
Ophiothrix sp.
Mytilus sp.
Necora sp.
B - Warm Temperate
Caprella sp.
Gammarus sp.
Ophitrix sp.
Haminoea sp.
Mytilus sp.
Paracentrotus sp.
C - Tropical
Atherinomorus sp.
Holothuria sp.
Ophiocoma sp.
Dascyllus sp.
Thalamita sp.
Modiolus sp.
Mortality (%)
Temperature (°C)
0
20
40
60
80
100
25
30
35
40
45
50

Supplementary figure S3. Wavelet analysis showing the dominant periodicities of the oxygen and temperature time series in the three study sites. The horizontal axis represents time and the vertical axis represents the periodicity of the corresponding oscillation. The paler area is the cone of influence, where results should not be interpreted due to biases connected to edge effects, and the thick black contour is the 5% significance level.

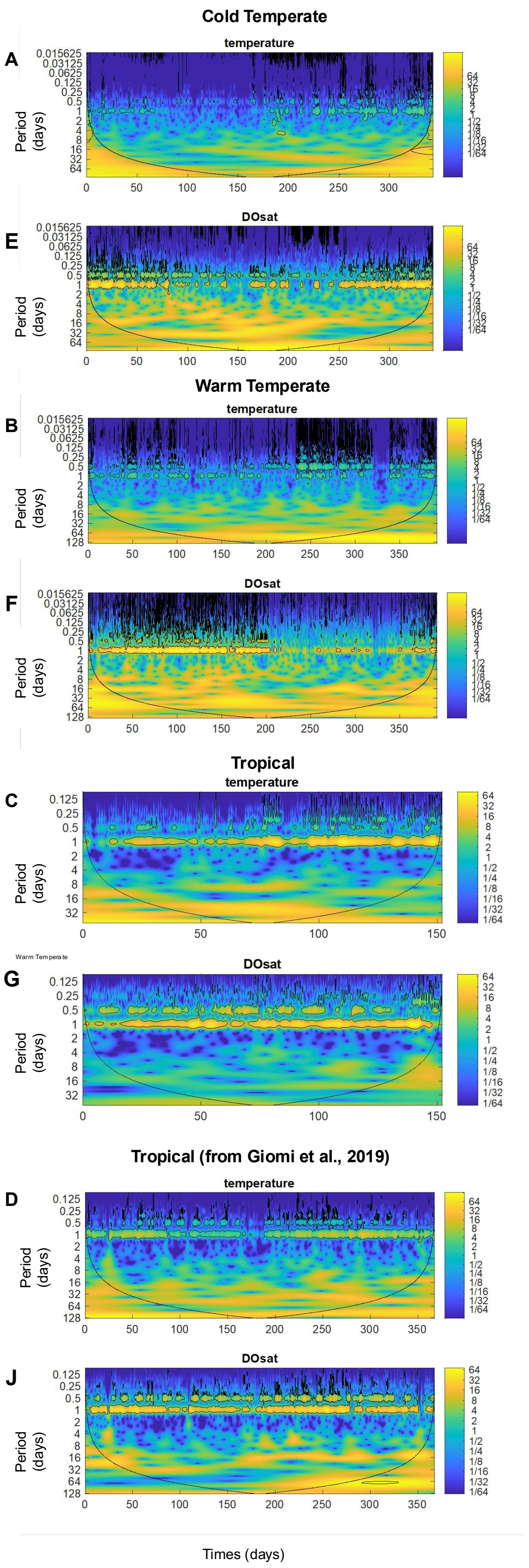
Cold Temperate
temperature
A
Period (days)
E
DOsat
Period (days)
Warm Temperate
temperature
B
Period (days)
F
DOsat
Period (days)
Tropical
temperature
C
Period (days)
Warm Temperate
G
DOsat
Period (days)
Tropical (from Giomi et al., 2019)
temperature
D
Period (days)
J
DOsat
Period (days)
Times (days)

Supplementary figure S4. (A-D) Seawater temperature in the Cold Temperate, Warm Temperate and Tropical coastal sites.

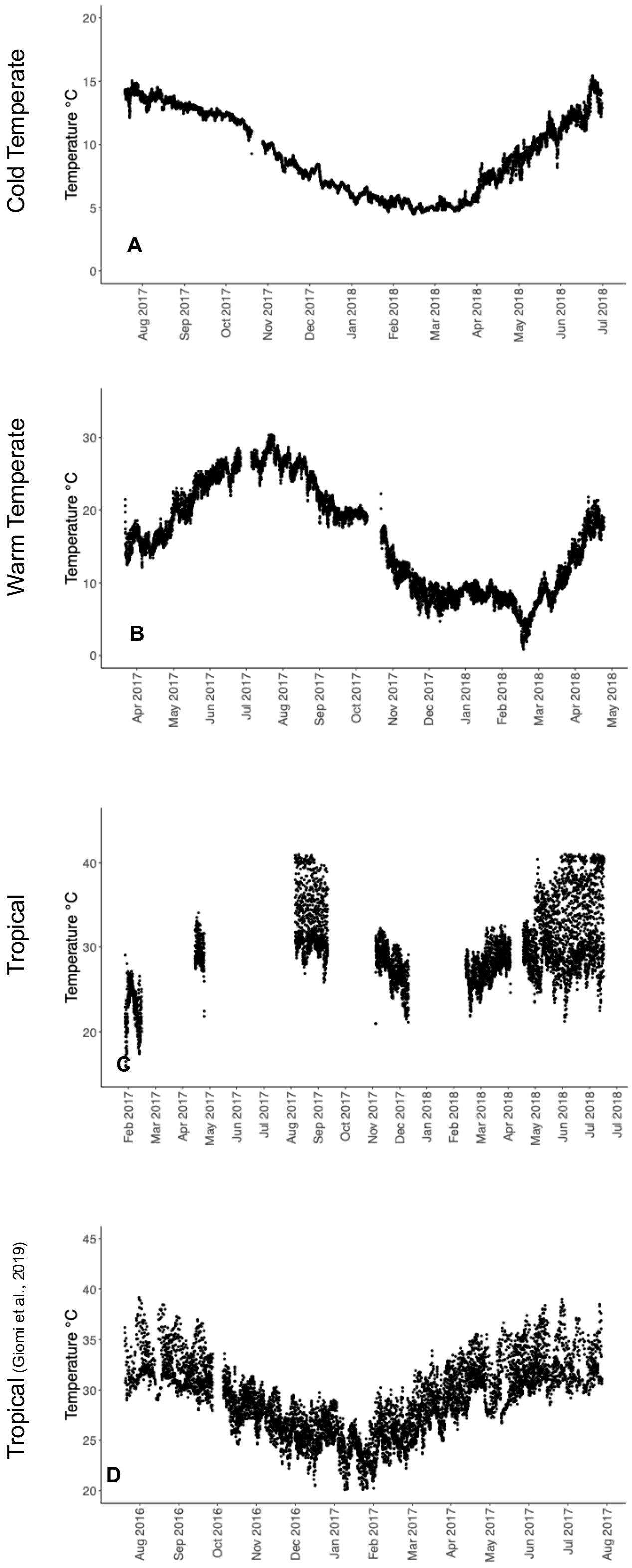

Supplementary Table S6. Statistical analysis for the comparison of LT50 between 97% and 140% of water saturation.

Location	Species	Oxygen sSaturation Treatment	LT50 Estimate (°C)	Std. Error	t-value	p-value
Cold Temperate	Echinus sp.	Normal Saturation	27.32	0.01	4282.45	<0.001
Cold Temperate	Echinus sp.	Supersaturation	28.78	0.01	4646.30	<0.001
Cold Temperate	Ostrea sp.	Normal Saturation	35.72	0.11	313.75	<0.001
Cold Temperate	Ostrea sp.	Supersaturation	36.37	0.08	461.66	<0.002
Cold Temperate	Ophiothrix sp.	Normal Saturation	30.35	0.01	2293.73	<0.002
Cold Temperate	Ophiothrix sp.	Supersaturation	31.79	0.03	1260.76	<0.001
Cold Temperate	Mytlus sp.	Normal Saturation	35.81	0.03	1132.57	<0.002
Cold Temperate	Mytlus sp.	Supersaturation	36.29	0.01	2487.57	<0.001
Cold Temperate	Necora sp.	Normal Saturation	30.86	0.16	198.66	<0.001
Cold Temperate	Necora sp.	Supersaturation	33.11	0.08	436.47	<0.001
Warm Temperate	Caprella sp.	Normal Saturation	34.44	0.02	1397.32	<0.001
Warm Temperate	Caprella sp.	Supersaturation	35.20	0.03	1405.96	<0.002
Warm Temperate	Gammarus sp.	Normal Saturation	36.45	0.05	677.54	<0.001
Warm Temperate	Gammarus sp.	Supersaturation	36.74	0.04	1023.59	<0.001
Warm Temperate	Haminoea sp.	Normal Saturation	39.07	0.20	194.57	<0.001
Warm Temperate	Haminoea sp.	Supersaturation	40.09	0.21	190.26	<0.001
Warm Temperate	Mytlus sp.	Normal Saturation	39.27	0.01	5502.75	<0.002
Warm Temperate	Mytlus sp.	Supersaturation	40.30	0.01	5658.16	<0.001
Warm Temperate	Ophitrix sp.	Normal Saturation	33.98	0.02	1723.51	<0.001
Warm Temperate	Ophitrix sp.	Supersaturation	34.34	0.02	1575.63	<0.001
Warm Temperate	Paracentrotus sp.	Normal Saturation	34.94	0.01	2336.75	<0.001
Warm Temperate	Paracentrotus sp.	Supersaturation	35.81	0.02	1825.40	<0.001
Tropical	Dascyllus sp.	Normal Saturation	37.47	0.07	516.20	<0.001
Tropical	Dascyllus sp.	Supersaturation	39.17	0.05	827.01	<0.001
Tropical	Hatherinomorus sp.	Normal Saturation	40.13	0.08	509.36	<0.001
Tropical	Hatherinomorus sp.	Supersaturation	41.38	0.09	439.26	<0.002
Tropical	Modiolus sp.	Normal Saturation	41.97	0.05	833.10	<0.001
Tropical	Modiolus sp.	Supersaturation	44.37	0.08	587.35	<0.001

Tropical	Ophiocoma sp.	Normal Saturation	43.05	0.02	2541.81	<0.001
Tropical	Ophiocoma sp.	Supersaturation	44.21	0.02	2810.43	<0.001
Tropical	Holothuria sp.	Normal Saturation	37.31	0.11	329.15	<0.001
Tropical	Holothuria sp.	Supersaturation	39.90	0.14	293.00	<0.001
Tropical	Thalamita sp.	Normal Saturation	40.76	0.05	850.49	<0.001
Tropical	Thalamita sp.	Supersaturation	43.98	0.04	1027.10	<0.002
